# Cytotoxic T-lymphocyte elicited therapeutic vaccine candidate targeting cancer against MAGE-A11 carcinogenic protein

**DOI:** 10.1042/BSR20202349

**Published:** 2020-12-02

**Authors:** Neeraj Kumar, Damini Sood, Aditya Gupta, Niraj Kumar Jha, Pallavi Jain, Ramesh Chandra

**Affiliations:** 1 Department of Chemistry, University of Delhi, Delhi 110007, India; 2 Department of Chemistry, SRM Institute of Science and Technology, Delhi-NCR Campus, Ghaziabad, Uttar Pradesh 201204, India; 3 Thomas H. Gosnell School of Life Sciences, Rochester Institute of Technology, Rochester, NY 14623, United States of America; 4 Department of Biotechnology, School of Engineering & Technology (SET), Sharda University, Greater Noida, Uttar Pradesh 201310, India

**Keywords:** Cytotoxic T-cell epitope, Full Flexibility Dynamics, Immunoinformatics, Molecular Docking Simulation, Peptide Rearrangements

## Abstract

Immunotherapy is a breakthrough approach for cancer treatment and prevention. By exploiting the fact that cancer cells have overexpression of tumor antigens responsible for its growth and progression, which can be identified and removed by boosting the immune system. *In silico* techniques have provided efficient ways for developing preventive measures to ward off cancer. Herein, we have designed a potent cytotoxic T-lymphocyte epitope to elicit a desirable immune response against carcinogenic melanoma-associated antigen-A11. Potent epitope was predicted using reliable algorithms and characterized by advanced computational avenue CABS molecular dynamics simulation, for full flexible binding with HLA-A*0201 and androgen receptor to large-scale rearrangements of the complex system. Results showed the potent immunogenic construct (KIIDLVHLL), from top epitopes using five algorithms. Molecular docking analyses showed the strong binding of epitope with HLA-A*0201 and androgen receptor with docking score of −780.6 and −641.06 kcal/mol, respectively. Molecular dynamics simulation analysis revealed strong binding of lead epitope with androgen receptor by involvement of 127 elements through atomic-model study. Full flexibility study showed stable binding of epitope with an average root mean square deviation (RMSD) 2.21 Å and maximum RMSD value of 6.48 Å in optimal cluster density area. The epitope also showed remarkable results with radius of gyration 23.0777 Å, world population coverage of 39.08% by immune epitope database, and transporter associated with antigen processing (TAP) affinity IC_50_ value of 2039.65 nm. Moreover, *in silico* cloning approach confirmed the expression and translation capacity of the construct within a suitable expression vector. The present study paves way for a potential immunogenic construct for prevention of cancer.

## Introduction

Cancer refers to a diseased condition in which a group of cells exhibit abnormal growth and division, which spreads to the neighboring tissues, forming a malignant tumor. It has aroused much concern in the last few decades, causing over 1.7 billion deaths through 2012 [1]. This number is expected to increase by ∼70% over the next two decades [2]. Cancer treatments comprise cancer immunotherapy, chemotherapy, radiotherapy, and surgery [3]. Among these, the only one targeting the quiescent cancer stem cells for a cure for cancer is immunotherapy [4]. Cancer immunotherapy theorizes the identification of cancer cells through immune cells. Therefore, only a vaccine capable of eliciting T-cell response can qualify as an immunotherapy candidate. However, tumor-induced immunosuppression is a problem which challenges this strategy [5–7]. Many different studies suggest that cancer antigens can elicit specific cellular and humoral immune responses. A successful alternative to prior existing vaccines is tumor antigenic peptide vaccines [8,9]. The positive contribution of peptide vaccines is also substantiated by clinical trials. However, challenges to the design and development of these vaccines are posed by potency, characterization of antigens, desired action of the vaccine, and delivery method [10]. One of the novel approaches used to design potential vaccine candidates is employing immunoinformatics, which includes the retrieval of tumor responsible proteins and the identification of B-cell and T-cell epitopic regions by employing immunological databases. The predicted epitopes can then be optimized with molecular docking simulation approaches. In the last two decades, cancer immunotherapeutics have become an important approach for the prevention of cancer. Tumor protein-derived antigenic epitopes have been extensively used to trigger the tumor-specific cytotoxic T lymphocytes (CTLs). Importantly, antigen-specific immune responses induced by MAGE (melanoma antigen)-derived antigenic peptides have been proven to be highly efficacious in the treatment of various type of tumors [11–13]. However, there are no studies to report the MAGE-A11 CTL epitopes as antitumor vaccine. Also, there were fewer reports to screen the MAGE immune signatures (epitopes) with potential immunological parameters, along with a stable and conformational flexible binding assessment of vaccine with target receptors. Hence, the present study was focused to examine the MAGE-A11 sequence with vital immune properties, binding affinity with membrane receptors, and prolonged large flexible binding through molecular dynamics simulation.

The MAGE is a family of cancer antigens and is a popular candidate in cancer immunology. The MAGE-A family is part of a group of the most commonly researched antigens with 12 known genes. It exhibits maximum expression in various cancers such as lung cancer, breast cancer, urothelial malignancies, oral squamous cell carcinoma, esophageal carcinoma, hematopoietic malignancies and cutaneous melanoma [14–19]. The MAGE-A11 is usually involved in the control of the androgen receptor signaling network. It can increase the transcriptional activity of the androgen receptor by chemical mechanisms that include phosphorylation of Thr^360^ and ubiquitination of Lys^240^ and Lys^245^ induced by the Epidermal Growth Factor [20,21]. Abnormally high quantities of MAGE-A11 are associated with high expressions of the androgen receptor protein in prostate cancer [22]. The increased expression of MAGE-A11 has a direct association with the progression of prostate cancer. Elevated levels of MAGE-A11 are also reported in breast cancer [23,24]. Therefore, in this work, the MAGE-A11 protein has been used as a target tumorigenic protein for designing potential peptide vaccine candidates.

With the development of immunoinformatics tools, it has become comparatively easier to predict the potent CTLs epitopes, which in turn, helps us to design a vaccine for cancer with a higher specificity to target protein receptor. *In silico* methods have also been performed for the identification of peptide vaccines for MAGE-A4, MAGE-A12, and many other tumorigenic proteins [25]. Also, in many viral diseases and case studies for dengue virus, herpes virus, acute encephalitis etc, similar immunological approaches have been employed to design the potential vaccine and successfully implemented [26,27].

Using *in silico* tools, the time needed for *in vitro* experiments can be reduced enormously. *In vitro* approaches to vaccine design and development include structural techniques like NMR, X-ray crystallography, and infrared spectrometry and other functional immunoassays, which are required to predict the epitopes bound to HLA alleles. These *in vitro* identification and optimization experiments are tedious and too expensive to define reliable peptide vaccine candidates. Hence, HLA-A*0201 protein-specific epitopes have been explored and identified. The HLA-A*0201 is an MHC I allele, which plays a central role in our immune systems. Major Histocompatibility Complex (MHC) molecules are very stable (half-life up to 10 h) and have a polymorphic nature. This allows them to bind to a vast array of foreign peptides [28]. Based on the accuracy of immunoinformatics tools, we predicted highly reliable T-cell epitopes to MAGE-A11. The accuracy was measured via ligand–protein interaction studies. Molecular docking and molecular dynamics simulation analyses were performed to investigate the binding of the epitopic region of tumor proteins with specific receptor proteins and also to analyze the molecular interactions between the epitope and target receptor proteins.

## Results

The present study aims to use advanced immunoinformatics approaches to identify the potential epitopic region of the MAGE-A11 tumorigenic protein, which could be used to elicit and strengthen the immune response to fight against the overexpressing tumor antigens. The complete sequence analysis of the potent tumorigenic protein, reliable prediction of CTLs, high binding affinity with membrane receptor, and prolonged stable binding could help in the possible identification of promising antigenic cancer vaccine candidate. These immune parameters were employed to define the potent CTL epitope to pave a vaccine candidate against cancer.

### MAGE-A11 sequence retrieval and physicochemical analysis

MAGE-A11 is reported as a proto-oncogene, and an increased level is intrudingly associated with many cancer types including lung cancer and prostate cancer, and considered to be potential targets for transcriptional cell cycle control and immunotherapies [29,30]. The amino acid sequence of MAGE-A11 (accession no. NP_005357.2), which has a size of 429 amino acids and molecular weight of 48129.24 Daltons, was retrieved. In sequence analysis through BLAST algorithm, we found MAGE protein consists of a conserved domain present in significant classes of the MAGE family. It is melanoma-associated antigen family N terminal (115–204), and these are tumor rejection antigens, which are expressed on HLA-A1 of tumor cells, and they are recognized by CTLs. MAGE family domain (243–397) was found to express in a wide variety of tumors. Moreover, theoretical pI and aliphatic index were computed to be 4.69 and 79.30, respectively. The GRAVY score was −0.431, which exhibited the hydrophilic nature of the protein sequence. The protein sequence was found to possess an estimated half-life of 30 h in mammalian reticulocytes *in vitro*. These results signified the sustainability of the antigenic sequence and can be further explored.

### Identification of potential CTL epitopes

The CTL epitopes for the MAGE-A11 tumorigenic protein were investigated. The peptide antigenic sequence binds to the MHC molecules and appears on the cell surface of the infected cell. CTLs recognize the peptide–protein complex and kill the infected cells. CTL epitopes are rendered as a potential candidate in the scope of *in silico* cancer vaccine design. The potent CTL epitopes were identified using the five different algorithms mentioned above (Rankpep, Bimas, NetMHC 4.0, Syfpeithi, and MHCPred) (Table 1). After that, the outcome of all five algorithms was superimposed to find the common consensus CTL epitopes, which could be potent epitope candidates for cancer vaccine designing. Among the top ten epitopes from all five servers, we found eight common epitopic sequences: ILHDKIIDL, KIIDLVHLL, KVLEYIANA, VMWEVLSIM, VMWEVLSIM, VLWGPITQI, FLWGPRAHA, and GLLIIVLGV (Table 2). These epitope sequences were further analyzed for their antigenicity, immunogenicity and transporter associated with antigen processing (TAP) affinity.

**Table 1:** The MHC-1 restricted CTL epitopes of the MAGE-A11 tumorigenic protein sequences, predicted using five different algorithms

Epitope rank	Rankpep	BIMAS	NetMHC 4.0	SYFPEITHI	MHCPred
1	VLWGPITQI	GLLIIVLGV	**KIIDLVHLL**	GLLIIVLGV	VLWGPITQI
2	ILHDKIIDL	FLFGEPKRL	KVLEYIANA	ILHDKIIDL	**KIIDLVHLL**
3	FLFGEPKRL	ILHDKIIDL	VLWGPITQI	**KIIDLVHLL**	KVLEYIANA
4	FLWGPRAHA	VLWGPITQI	GLLIIVLGV	VLWGPITQI	VMWEVLSIM
5	VMWEVLSIM	VMWEVLSIM	VMWEVLSIM	LLIIVLGVI	FLFGEPKRL
6	GLLIIVLGV	FLWGPRAHA	FLFGEPKRL	ALREEGEGV	DLTRVIMPL
7	**KIIDLVHLL**	MQLLFGIDV	ILHDKIIDL	GLITKAEML	SIMGVYAGR
8	AMDAIFGSL	**KIIDLVHLL**	FLWGPRAHA	GLGCSPASI	PITQIFPTV
9	PITQIFPTV	KVLEYIANA	AMDAIFGSL	HLLLRKYRV	FLWGPRAHA
10	HLLLRKYRV	VLSIMGVYA	YVLVTSLNL	FLFGEPKRL	KMKVLEYIA

Top ten ranked predicted epitopes have been depicted with all five servers. The lead epitope is shown in bold.

**Table 2:** The most similar epitopes from five different algorithms are depicted here

S.No.	Epitopes	VaxiJen score	Antigenicity predictions	Binding affinity
1	KIIDLVHLL	1.3265	Probable antigen	−780.06 kcal/mol
2	ILHDKIIDL	0.8380	Probable antigen	−581.3 kcal/mol
3	KVLEYIANA	0.7691	Probable antigen	−597.7 kcal/mol
4	VMWEVLSIM	0.7083	Probable antigen	−748.6 kcal/mol
5	FLFGEPKRL	0.5558	Probable antigen	−635.1 kcal/mol
6	VLWGPITQI	0.1494	Probable non-antigen	N/A
7	FLWGPRAHA	0.0666	Probable non-antigen	N/A
8	GLLIIVLGV	−1.9171	Probable non-antigen	N/A

These were tested for their antigenicity characteristics using VaxiJen v2.0 server. Epitopes with their antigenicity scores are enlisted in the table.

### C-terminal cleavage efficiency and TAP affinity determination

The most similar epitopes from five different algorithms were further studied for their C-terminal cleavage efficiency, TAP affinity, and binding affinity to HLA*0201 receptor. The C-terminal cleavage efficiency and TAP transport capability were determined using the netCTLpan server. Obtained results showed that the lead epitope ‘KIIDLVHLL’ has a high cleavage efficiency with a weighted score of 0.952 on proteasomal C-terminal with cut-off value of 0.225; as per algorithm peptides with high score are more susceptible to proteasomal C-terminal cleavage required step for antigen processing (Table 3) [31]. Results also showed the high TAP efficiency of the lead epitope (1.120) with cut-off value of 0.025. The ubiquitin proteasomal cleavage system works for processing of the large size peptides/polypeptides into the unfolded protein and further hydrolyzes them into short peptides (8–10 amino acids) for antigen presentation to MHC Class-1 T cells to generate immune response [32].

**Table 3:** NetCTLpan predictions of lead epitope for HLA-A*02:01 allele

Peptide	MHC	TAP	Cle	Comb	% Rank
KIIDLVHLL	0.79200	1.12000	0.92523	1.02818	0.15
KVLEYIANA	0.76700	0.23600	0.91951	0.96799	0.30
VLWGPITQI	0.66900	0.73900	0.97585	0.90704	0.80
ILHDKIIDL	0.65000	0.88800	0.94766	0.88542	0.80
FLFGEPKRL	0.66200	0.96800	0.85336	0.87821	0.80
AMDAIFGSL	0.62300	1.05400	0.97232	0.86812	0.80
VMWEVLSIM	0.63100	0.49300	0.88705	0.84291	1.00
FLWGPRAHA	0.59700	0.60600	0.96299	0.79852	1.50
GLLIIVLGV	0.62900	0.22700	0.65914	0.78298	1.50
YVLVTSLNL	0.48700	1.14500	0.96881	0.73361	2.00
MQLLFGIDV	0.52400	0.44800	0.84648	0.72566	2.00

Moreover, the TAP affinity of the lead epitope was also assessed using another server, Tapreg. The predictive performance of the lead epitope TAP transport affinity model was evaluated to be 2039.65 nm (IC_50_ value), under ten-fold cross-validation experiments measured using Pearson’s correlation coefficients. Also, lead epitope was found to bind with the highest binding affinity. These significant results suggested that the lead epitope can also directly move in the endoplasmic reticulum to the MHC-1 receptor with its top TAP and MHC binding affinity.

### Antigenicity analysis and selection of epitope

We identified the antigenicity efficacy and immunogenicity of the shortlisted epitopes, using the VaxiJen v2.0 server. The VaxiJen server applies a new alignment-independent approach that is based on the auto cross-covariance transformation of protein sequences into uniform vectors of principal amino acid properties. Depending on the target organisms (bacterial, viral, and tumor protein datasets), the accuracy of the server is calculated to be 70–89%. The CTL epitope (KIIDLVHLL) resulted in the highest Vaxijen score of 1.3265. A high vaxijen score (threshold value 0.4), suggests that the predicted epitope has a higher antigenicity and is hence a potential candidate for vaccine development. The lead epitope ‘KIIDLVHLL’ was further evaluated for its detailed interaction with HLA-A*0201 through molecular docking.

### HLA-A*0201 protein structure retrieval and structural analysis

**Figure 1:**
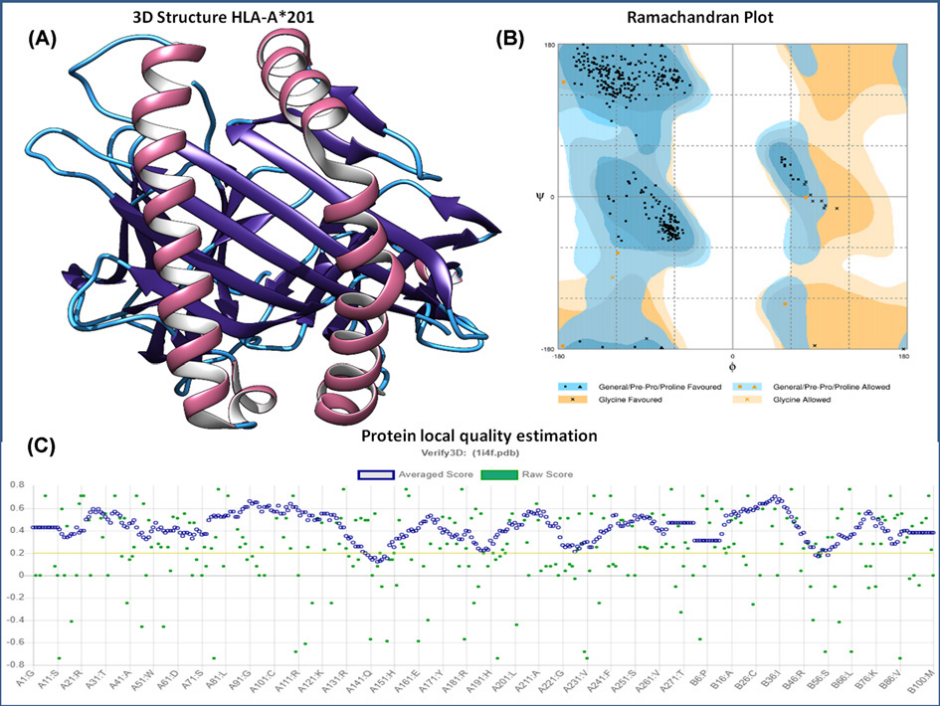
HLA-A*0201 structure and its assessment (**A**) Three-dimensional structure of HLA-A*0201 receptor protein retrieved from PDB. (**B**) HLA-A*0201 protein structure characterization: Ramachandran plot analysis showed the 98.4% of the structure residues are in the favorable region and 1.6% of residues in the additionally allowed region, suggesting the good quality of the structure. (**C**) Structural local quality estimation showing minimal deviation throughout the protein.

The 3D HLA-A*0201 protein complex was retrieved from the RCSB Protein Data Bank (PDB: 1I4F) to perform the molecular docking analyses. It was a crystal structure of HLA-A*0201/MAGE-A4–peptide of resolution 1.4 Å. HLA-A*0201 three-dimensional structure was extracted from the conjugated structure. The protein HLA-A*0201 is made up of two chains, chain-A is of 275 amino acids, and β-2 microglobulin is of 100 amino acids. The extracted HLA-A*0201 protein structure was analyzed for its stereochemical properties with a Ramachandran plot. The 3D protein structure was also evaluated with the Verify3D, Prosa, and Errat analysis tools. The Ramachandran plot of the HLA-A*0201 protein structure showed that 98.4% of the residues are in the favorable region, and 1.6% of the residues are in the allowed region (Figure 1). The Prosa tool showed that the Z-score of the A-A*0201 protein is −9.36, which indicates the good overall quality of the protein structure. The Errat program showed a score of 97.493, the quality factors of structure, which suggests that the HLA-A*0201 structure has a high resolution. The Verify3D server showed that 97.00% of the residues had an averaged 3D-1D score ≥ 0.2 and that the protein structure followed the algorithm. The Ramachandran plot, Z-score, and ERRAT analyses confirmed the good quality of the structure of the HLA-A*0201 protein.

### Binding affinity analysis of the lead epitope with HLA-A*0201

**Figure 2:**
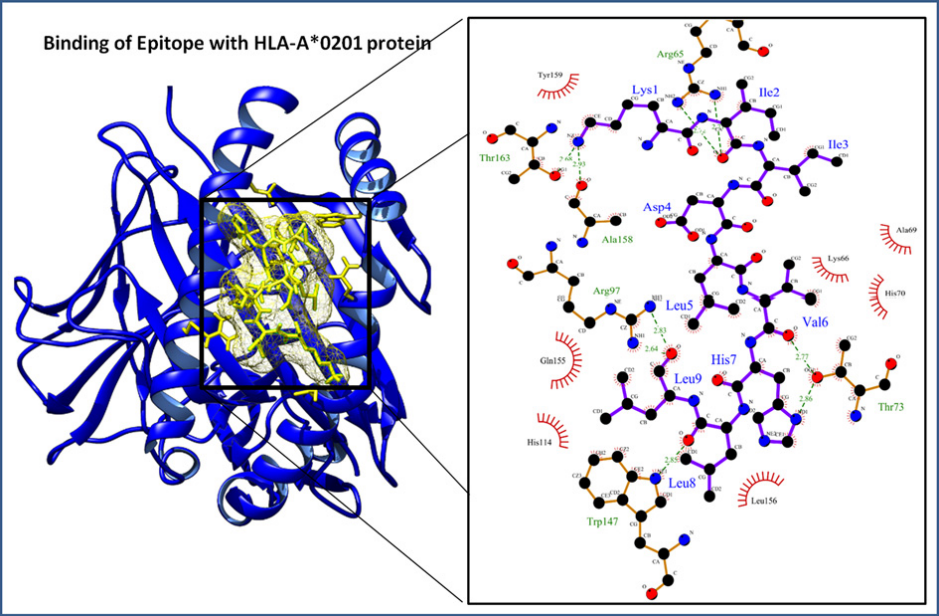
Docked protein complex of the lead epitope (yellow in color) and HLA-A*0201 protein (blue color ribbon cartoon view) Interaction in the binding groove and enlarged depiction of involved potential molecular interactions (Hydrophobic interactions and Hydrogen bonds) between the lead epitope and HLA-A*0201 protein at left side.

The binding affinity of the lead epitope with HLA-A*0201 was determined through molecular docking. The protein structures of HLA-A*0201 receptor protein were prepared before the docking using the Whatif server, and molecular docking was performed using the cluspro molecular docking interface. The molecular docking resulted in a docking score of −780.06 kcal/mol of the lead epitope with HLA-A*0201. The interacting complex was further evaluated using the Ligplot program to identify the molecular interactions between the lead epitope and the HLA-A*0201 protein receptor. The lead epitope was found to have strong molecular interactions with nine hydrogen bonds and various hydrophobic interactions (Figure 2). The hydrogen bonds were found between Lys^1^–Thr^163^ of bond length 2.68 Å, Lys^1^–Ala^158^ of bond length 2.93 Å, Ile^2^–Arg^65^ (NH1) of bond length 2.71 Å, Ile^2^–Arg^65^ (NH2) of bond length 2.72 Å, Val^6^–Thr^73^ of bond length 2.77 Å, His^7^ (ND1)–Thr^73^ (OG1) of bond length 2.86 Å, Leu^8^–Trp^147^ of bond length 2.85 Å, Leu^9^–Arg^97^ of bond length (NH1) 2.64 Å, and Leu^9^–Arg^97^ (NH2) of bond length 2.83 Å, of the lead epitope and HLA-A*0201 protein receptor. The lead epitope was also found to have strong hydrophobic interactions with Lys^66^, Ala^69^, His^70^, His^114^, Gln^155^, Leu^156^, and Tyr^159^ residues of the HLA-A*0201 receptor (Table 4).

**Table 4:** Lead epitope (KIIDLVHLL) molecular interaction analyses with HLA-A*0201 receptor

Epitope (9 amino acid)	Receptor protein	Binding affinity	Hydrogen bond and bond length	Hydrophobic interactions
KIIDLVHLL	HLA – A*02:01	−780.06 kcal/mol	Lys^1^ (NZ)–Thr^163^ (OG1): 2.68 Å	Lys^66^, Ala^69^, His^70^, His^114^, Gln^155^, Leu^156^, Tyr^159^
			Lys^1^ (NZ)–Ala^158^ (O): 2.93 Å	
			Ile^2^ (O)–Arg^65^ (NH1): 2.71 Å	
			Ile^2^ (O)–Arg^65^ (NH2): 2.72 Å	
			Val^6^ (O)–Thr^73^ (OG1): 2.77 Å	
			His^7^ (ND1)–Thr^73^ (OG1): 2.86 Å	
			Leu^8^ (O)–Trp^147^ (NE1): 2.85 Å	
			Leu^9^ (O)–Arg^97^ (NH1): 2.4 Å	
			Leu^9^ (O)–Arg^97^ (NH2): 2.83 Å	
	Androgen receptor	−641.36 kcal/mol	Lys^1^ (NZ)–Glu^678^ (O): 2.69 Å	Ala^679^, Pro^682^, Gly^683^, Val^684^, Val^685^, Trp^751^, Arg^752^, Thr^755^, Tyr^763^, Pro^766^, Pro^801^, Phe^804^
			Lys^1^ (NZ)–Glu^681^ (O): 2.73 Å	
			His^7^ (ND1)–Asn^756^ (OD1): 2.57 Å	
			His^7^ (O)–Asn^756^ (ND2): 2.73 Å	
			Leu^9^ (O)–Glu^711^ (NE2): 2.87 Å	

The table depicted the strong interaction with nine hydrogen bonds and strong hydrophobic interactions. Also, lead epitope was docked with the androgen receptor and showed strong interactions with five hydrogen bonds and hydrophobic interactions.

Moreover, the compactness of the epitope- HLA-A*0201 protein complex was calculated by the radius of gyration of the complex. The radius of gyration defines the overall spread of the molecule and describes the root mean square distance of atoms from their common center of gravity. A radius of gyration (Rg) score of 23.0777 Å showed the stable globular protein complex of HLA-A*0201 and lead epitope.

### Optimization of lead epitope by binding analysis with membrane-specific androgen receptor

**Figure 3:**
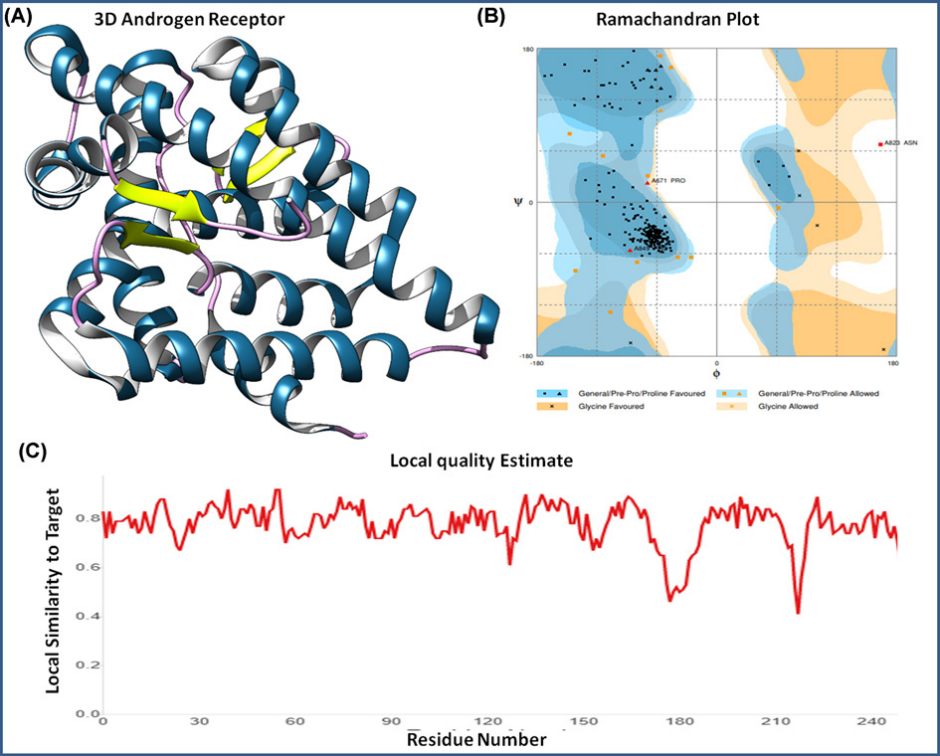
Androgen receptor and its structural analysis (**A**) Three-dimensional structure of androgen receptor retrieved from PDB. (**B**) Ramachandran plot analysis of androgen receptor; 94% residues in favorable region, 4.2% residues in allowed region and 1.2% residues in outlier region. (**C**) Local protein sequence quality estimation of the androgen receptor.

MAGE protein is involved in cancer signaling pathways by interacting with the androgen receptor during cancer growth and progression. Hence, the epitope sequence was studied for its interaction with the androgen receptor using a molecular docking approach. The androgen receptor was downloaded from the PDB (PDB ID: 1E3G). The crystal structure was determined by X-Ray Diffraction of resolution 2.4 Å. It was found in co-crystal conjugation with ligand metribolone (R1881), which was removed from the 3D of androgen receptor structure to perform the molecular dynamics studies. It was analyzed by Ramachandran plot for stereochemical properties and found optimal with 94% percent of residues in the favorable region, 4.2% residues in allowed region, and only 1.2% residues in the outlier region. Also, local quality estimation of protein showed the stability of the 3D structure with minimal fluctuations in residues (Figure 3).

**Figure 4:**
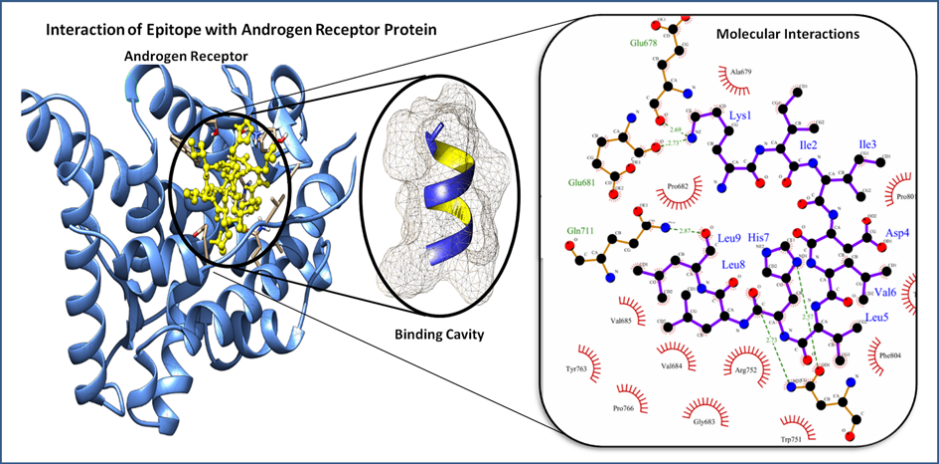
Figure depicts the docked protein complex of the lead epitope (KIIDLVHLL) yellow in color and androgen receptor protein in blue color ribbon cartoon (right side), and (left side) depiction of involved molecular interaction (Hydrophobic interactions and Hydrogen bonds) of the epitope to the HLA-A*0201 protein

The prepared androgen receptor was docked with a lead epitope, which resulted in a docking score of −641.36 kcal/mol (Figure 4). The androgen receptor–lead epitope protein complex showed five hydrogen bonds and multiple hydrophobic interactions. The hydrogen bonds were formed between Lys^1^–Glu^678^ of bond length 2.69 Å, Lys^1^–Glu^681^ of bond length 2.73 Å, His^7^–Asn^756^ (OD1) of bond length 2.57 Å, His^7^–Asn^756^ (ND2) of bond length 2.73 Å and Leu^9^–Glu^711^ of bond length 2.87 Å of the epitope residues and androgen receptor, respectively. The lead epitope has strong hydrophobic interactions with the residues of Ala^679^, Pro^682^, Gly^683^, Val^684^, Val^685^, Trp^751^, Arg^752^, Thr^755^, Tyr^763^, Pro^766^, Pro^801^, and Phe^804^ residues of the androgen receptor. These hydrogen bonds, along with the hydrophobic interactions, suggested the strong binding of the lead epitope with the HLA-A*0201 receptor protein.

### Molecular dynamics and peptide dynamics of epitope using CABS simulation

Lead epitope binding to the androgen receptor was further analyzed using the molecular dynamics simulation, which provided insight into the large-scale binding conformations of the epitope. The near-native dynamics for 10-ns system simulation was performed employing the CABS (for all atoms, explicit water using the potential force fields) for conformation flexibility analysis. CABS interface is a coarse-grained protein simulation-based algorithm that assesses the target protein–peptide complex dynamics. It involved the knowledge-based statically potentials responsible for solvent effects in implicit fashions, via short-range conformational based on protein sequence, statics of pairwise interactions of side chains, and hydrogen bonds of cluster models. Conformational analysis performed was regulated by the Monte Carlo schemes to simulate by random series of small moves, and compilation results in the evolution of protein dynamics [33]. The 3D protein structure of the androgen receptor and lead epitope were put as input data for molecular dynamics simulation. CABS followed the multistep filtration of generated models and clustered them with atom representations (element involvement) of interacting complex and followed by local optimization of top models.

**Figure 5:**
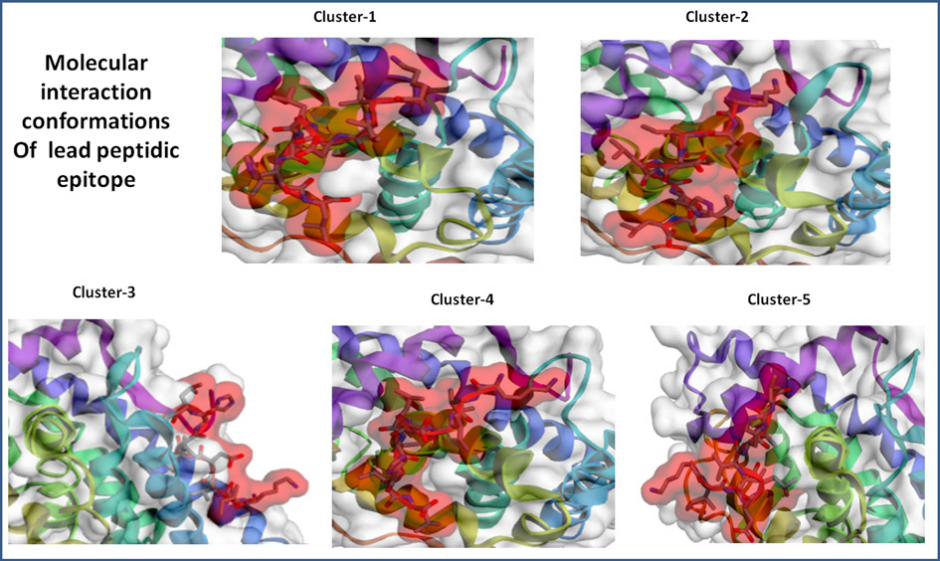
Top scored cluster models of the interacting molecular complex system of lead epitope and androgen receptor with minimal average RMSD values and involved elements in stable interaction of the system Among all clusters, cluster-1 showed the most substantial interaction with the involvement of a high number of elements and minimal fluctuations in average RMSD.

**Figure 6:**
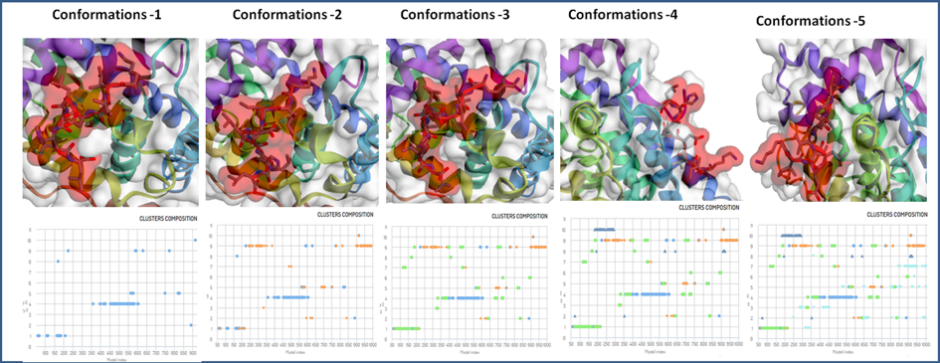
Binding conformations and Cluster composition of top five clusters of lead epitope and androgen receptor with minimal average RMSD values and involved elements in stable interaction

The epitope peptide was analyzed for rearrangement and flexibility for 50 simulation cycles. Resulting 10000 models, the top five trajectories were picked of the strong interaction. Five clusters were found to interact in close proximity of target protein androgen receptor with high energy minimization (Figure 5). Among these five clusters, cluster-1 has stable interaction with the involvement of a large number of 127 elements (Cluster consisted of grouped models) and average root mean square deviation (RMSD; Cα RMSD score between the grouped models in a cluster) 2.21 Å and maximum RMSD value of 6.48 Å in the cluster density of 57.418 (Clusters density is the number of elements in cluster/average RMSD between cluster elements). Moreover, Cluster-2 also depicted the stable interaction with involvement of large number of 111 elements (Cluster consisted of grouped models) and average RMSD 2.95 Å in the cluster density of 37.588, however less strong than cluster-1. Other cluster-3, Cluster-4 and Cluster-5 showed the involvement of 11079 and 102 elements, average RMSD 4.40, 4.51 and 6.77, cluster density of 24.979, 17.493 and 15.065 (Figure 6). The critical molecular dynamics analysis of the simulated clusters elucidated the closest and stable binding of lead epitope–receptor complex and among them Cluster-1 showed the strongest interaction through molecular dynamics simulation analyses.

**Figure 7:**
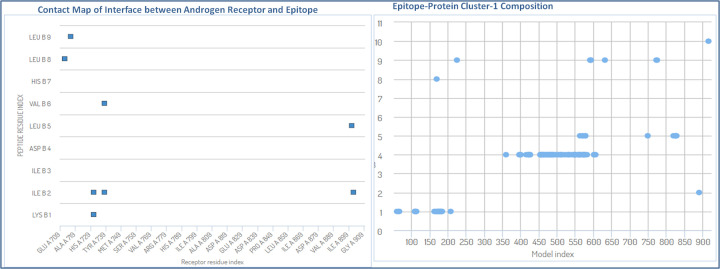
Contact map analysis of lead epitope and androgen receptor, depicting the involved amino acids in the interactions and its model (cluster-1) elements involvements in interactions through the atomic model study

The critical molecular dynamics analysis of the simulated cluster elucidated the binding residues of the complex and reconstruction of the stable complex system. The contact map analysis showed the pairs of peptide and residues closer than 3.0 Å in peptide–androgen complex are Val^901^ (A)–Leu^5^ (B), Asp^731^ (A)–Ile^2^ (B), Leu^712^ (A)–Leu^8^ (B), Gln^902^ (A)–Ile^2^ (B), Gln^738^ (A)–Ile^2^ (B), Val^716^ (A)–Leu^9^ (B), Gln^738^ (A)–Val^6^ (B) and Asp^731^ (A)–Lys^4^ (B), where chain-A is indicating the androgen receptor protein and chain-B is showing the epitope chain (Figure 7). These data confirmed the potential roles of various amino acids in the interaction of epitope with the target androgen receptor. The full flexibility analysis suggested the stable interaction of the docked system. The understanding of large conformational binding of epitope and androgen receptor protein, also supported the conformational selection and recognization of epitopes, as binding partner selects the most favored conformation.

### Immune response simulation analysis

**Figure 8:**
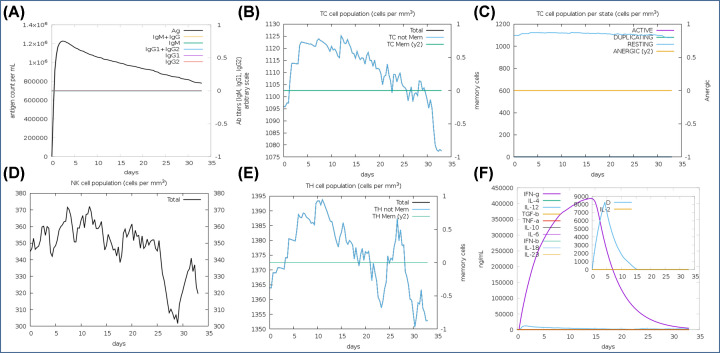
Immune response simulation analysis (**A**) High-level elevation of immunoglobulins by the administration of antigenic determinates at different time interval. (**B**) Cytotoxic T-cell population counts with an average level of 1102 cells per mm^3^. (**C**) Cytotoxic T-cell population at different states with the maximum at resting state. (**D**) Depiction of a high level of natural killer (NK) cell population. (**E**) Helper T-cell population at a different time interval. (**F**) High level of elicitation of interferons counts along with interleukins and other potential immune system regulators.

The immune response simulation analysis of lead antigenic determinant was conducted using the *in silico* method through C-ImmSim 10.1 server. We performed the 100 simulation steps at uniform speed with a selection of major classes of human leukocyte antigens. In results, we found, antigenic determinant elicits a robust immune response by evoking the potential immune response generating regulators. Administration of antigenic determinant elicited the high titer of primary antibodies (IgM, IgG1, IgG2) in the early days and then slowly decreased till 30 days. Importantly, it showed the elicitation of cytotoxic T cells up to the maximum 1125 cells per mm^3^ with an average optimal level elevation of 1102 cells per mm^3^; and then gradually decreases after 22–23 days of insertion of the vaccine candidate. Also, level remained high throughout the resting state, analyzed by static simulation computation (Figure 8). The B-cell population was also found to depict the prolonged the high response with an average of 472 cells per mm^3^ to a long time and further helped to evoke memory cells to strengthen immunity against the re-infection by memorizing the immune system. Besides, we found it to stimulate the helper T cells up to a maximum level of 1394 cells per mm^3^ with an average elevation of 1373 cells per mm^3^, along with other potential immune response generator regulators, including the interferons, natural killer cells, and interleukins. Obtained results depicted the potency of designed antigenic determinant to elicit a robust immune response against the tumorigenic state.

### Structural and conformational stability and world population coverage analysis of lead epitope

**Figure 9:**
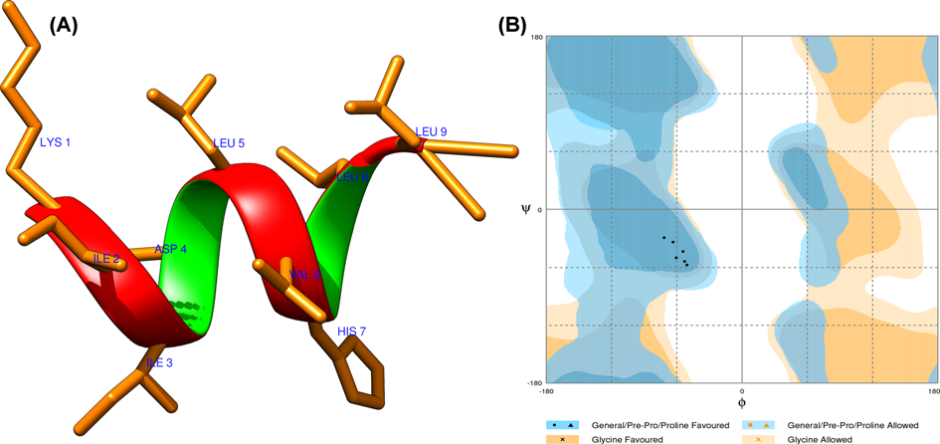
The lead epitope structural assessment (**A**) Three-dimensional structure of lead epitope (KIIDLVHLL), designed using the Pepfold 3.0. (**B**) Ramachandran plot of the lead epitope analysis, showed the 92.6% of the structure residues are in the favorable region, suggesting the good quality of the structure.

The physicochemical properties of the epitope were first calculated. The theoretical isoelectric point value and molecular weight of the lead epitope were computed to 1063.50 Daltons and 7.09, respectively, which indicated the nearly neutral character. The instability index value is computed to be −0.54, which suggests that the epitope is a stable protein. An estimated aliphatic index value of 248.89, indicates the epitope is thermostable, as the high aliphatic value suggests the more thermostable protein. Epitope has 1.3 h estimated half-life in mammalian reticulocytes through *in vitro* approach. The 3D structure of the lead epitope was designed by Pepfold 3.0 and analyzed using the Chimera molecular modeling suite. The 3D structure was optimized by using the Procheck and Rampage servers, which examine the structure through the Ramachandran plot for stereochemical properties. Procheck server showed that the epitope has 92.60% residues in the φ–ψ backbone favored region, and Rampage server also showed tertiary structure in the allowed region. Both servers signified the stability of the epitope structure (Figure 9). Also, the lead epitope was assessed for its cellular localization, which showed that it is majorly localized in the nuclear and mitochondrial region and with fewer ratios localization in cytoplasmic, extracellular plasma membrane, and other regions of the cell, by using the schemes employing amino acid composition, di-peptide/partitioned occurrence and sequence composition based on physicochemical properties (Table 5). Furthermore, the epitope’s world population coverage was determined by the Immune Epitope Database (IEDB). The lead epitope was found to have optimal world population coverage of 39.08%, and it as a promising cancer vaccine candidate.

**Table 5:** Cellular localization analysis of lead epitope

CELLO Results		
SVM	Localization	Reliability
Amino acid Comp.	Nuclear	0.345
N-Peptide Comp.	Mitochondrial	0.423
Partitioned seq. Comp.	Nuclear	0.418
Physico-chemical Comp.	Mitochondrial	0.444
Neighboring seq. Comp.	Mitochondrial	0.883
CELLO PREDICTIONS	Mitochondrial	2.049
	Nuclear	1.201
	Chloroplast	0.624
	Cytoplasmic	0.505
	Extracellular	0.361
	Plasma membrane	0.133
	Peroximal	0.034
	ER	0.025
	Lysosomal	0.019
	Cytoskeletal	0.018
	Vacuole	0.017
	Golgi	0.015

### *In silico* cloning expression analysis

**Figure 10:**
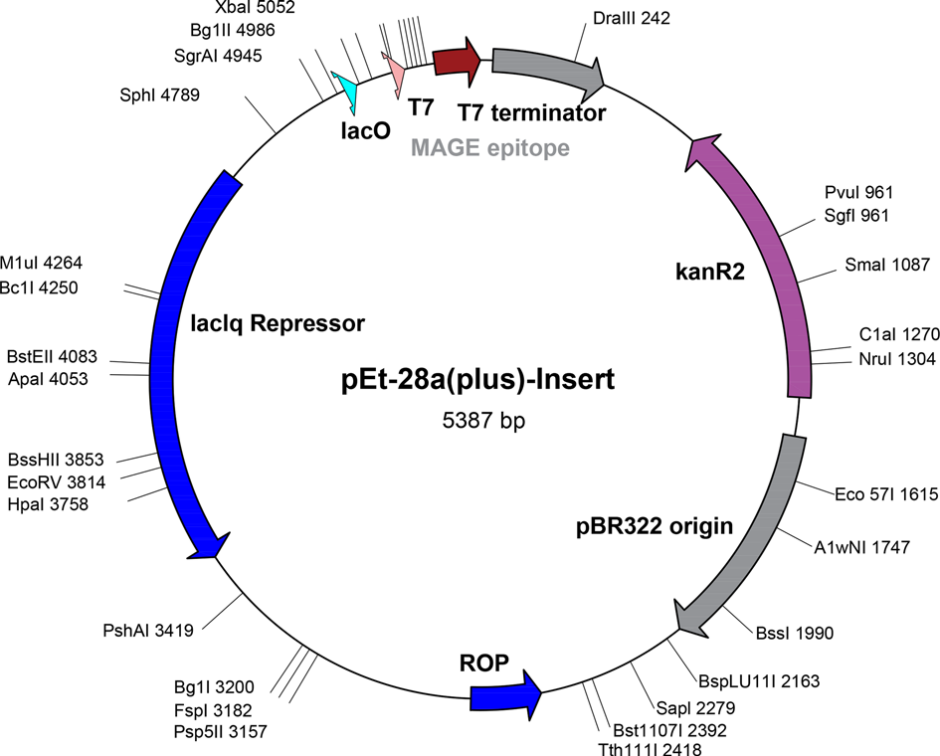
*In silico* cloning expression analysis of lead epitope

*In silico* cloning was employed to evaluate the cloning and expression ability of the lead epitope for vaccine development, within the suitable expression vector, using the Codon usage wrangler server. Reverse translation generated the cDNA sequence ‘AAGATCATCGACCTGGTGCACCTGCTG,’ which was further analyzed for codon optimization. Codon optimization depicted the high GC contents (60.7%) of the DNA construct of the lead epitope, which reflected the stability of the sequence. Cloning expression also showed the high expression of epitope construct in plasmid vector p28a with a total size of 5387 base pairs (Figure 10).

## Materials and methods

### Sequence retrieval and physicochemical analysis of MAGE-A11

MAGE-A11, a tumor antigen whose overexpression leads to growth and progression of various cancer including cervical, lung, and prostate. The complete protein sequence of MAGE-A11 was retrieved from the National Centre for Biotechnology Information (NCBI) (http://www.ncbi.nlm.nih.gov) and was subjected to a physicochemical analysis, which includes the determination of instability index, molecular weight, aliphatic index, theoretical pI, amino acid composition and a grand average of hydropathicity by ProtParam (http://web.expasy.org/protparam/) [34].

### Prediction of potential CTL epitopes using five different algorithms

CTLs epitopes were predicted using five prediction servers based on various algorithms in order to predict more efficient and potent vaccine candidates. Most of the MHC-1 receptor binds to antigen ligand of size 9 amino acids with an optimal range of 8–10 amino acids, hence epitope of nanomer range were determined. These CTL epitope prediction servers were Rankpep, BIMAS, NetMHC 4.0, SYFPEITHI, and MHCPred. First, the Rankpep server was used, which used a PSSM-based algorithm and resulted in 32 peptide sequences with a binding threshold value of 64 for the interaction with the HLA alleles 0201. Rankpep covers all the HLA supertypes and results in potent nanomer epitopes peptide sequences. An analysis of the results indicates that >80% of these epitopes are among the top 2% of scoring peptides [35]. After that, the second server, BIMAS was used. BIMAS predicts peptide vaccine candidates on the basis of the half-life of the MHC-1 complex with the coefficient rank of the peptide [36]. Next, the NetMHC 4.0 server was accessed, which predicts candidates based on binding affinities using gapped sequence alignments fed to an artificial neural network [37]. After that, the SYFPEITHI tool was used. It searches the epitopic region on the basis of published motifs considering the amino acids at auxiliary and anchor positions. SYFPEITHI server identifies the epitopes on the basis of published motifs, considering the amino acids at anchor positions and most frequent amino acids [38]. At last, MHCPred was accessed. It uses the additive physicochemical properties to predict high binding affinity to the MHC and transporter associated with processing [39]. Among the predicted epitopes from all five servers; the common consensus sequences were obtained to avail the diverse and highly authentic epitopes.

### Selection of lead epitope by C-terminal cleavage efficiency, TAP affinity, and antigenicity

We have further analyzed the shortlisted epitopes for peptide C-terminal cleavage efficiency and TAP transport capability using the netCTLpan server. Also, the shortlisted epitopes were further evaluated for their antigenicity and immunogenicity characteristics. The ubiquitin proteasomal cleavage system works for processing of the large size peptides/polypeptides into optimal CTL peptide size range. Identified epitopes were evaluated for TAP affinity. TAP proteins transport the antigen protein to the target MHC molecule. A high TAP affinity is an essential characteristic of an efficient vaccine candidate. The netCTLpan server and Tapreg server were employed to analyze the TAP affinity of the predicted epitopes [40]. The antigenicity and pathogenicity of CTL epitopes were assessed by Vaxijen V2.0 (http://www.ddg-pharmfac.net/vaxijen/VaxiJen/VaxiJen.html). The Vaxijen server works by using auto cross-covariance transformation to differentiate between antigenic and non-antigenic epitopes [41]. After that, shortlisted epitopes were studied for their binding interaction with the HLA-A*0201 protein and androgen receptor, a MAGE-A11 protein-specific receptor present on the cell surface.

### HLA-A*0201 protein structure retrieval and structural analysis

The HLA-A*0201 structure was retrieved from the PDB (PDB ID: 1I4F) [42]. The HLA-A*0201 structure is present in conjugation with the MAGE-A4–peptide complex. Hence, the HLA-A*0201 structure was extracted using the Swispdbviewer tool. The HLA-A*0201 structure was evaluated for its stability using a Ramachandran plot. Structural protein stereochemistry was assessed by the Prosa tool. ERRAT was used for 3D structure verification of HLA-A*0201. The Ramachandran plot helps evaluate the φ–ψ bond distribution for the protein structures under evaluation. The Ramachandran plot was generated using the Rampage server (http://mordred.bioc.cam.ac.uk/∼rapper/rampage.php). The quality of the protein 3D structure was then analyzed using the Prosa web tool by examining the structure’s Z-score (the overall quality factor of structure) (https://prosa.services.came.sbg.ac.at/prosa.php) [43]. The Z-score defines the structure quality by determining whether the predicted protein lies within the range formed by the scores for experimental determined high-quality structure proteins of the same size. Further protein models were evaluated using Errat (http://nihserver.mbi.ucla.edu/ERRAT/). Errat is software that verifies protein structures determined by crystallography. Errat values are plotted as a function of the position of a sliding residue window. The Errat function is based on the statistics of non-bonded atom–atom interactions in the reported structure (compared with a database of reliable high-resolution structures) [44].

### Binding analysis of epitope with HLA-A*0201 receptor

The HLA-A*0201 receptor was prepared for molecular docking studies. The protein preparation included the removal of water molecules, any duplicated chains, or any other unwanted heteroatoms. After that, the 3D structure of the ligand lead epitope was predicted to perform the molecular docking assays. The PEP-FOLD peptide structure prediction server was used to predict the 3D structures of the epitope. PEP-FOLD is a *de novo* 3D structure prediction tool for peptides with sizes ranging from 9 to 36 amino acids [45]. PEP-FOLD on the basis of the Hidden Markov Models algorithm, predicts the 3D structures starting from the sequence of the protein by performing a series of simulations which ends with conformations that are most representative of energy and population.

After that, molecular docking was performed using the ClusPro docking server. Cluspro is an automated docking module that uses fast rigid-body protein–protein docking to analyze the interactions between the receptor protein, and the ligand [46]. ClusPro is based on three techniques: first, a fast Fourier transform correlation. Secondly, energy conformation clustering and the third is an assessment of cluster stability by Monte Carlo simulations. The docked epitope–protein complex was analyzed by Ligplot to determine the involved molecular interactions. Moreover, the Rg analysis was also checked to identify the compactness and stability of the docked interacting complex of the epitope and HLA-A*0201 protein receptor.

### Epitope optimization by binding with homing membrane androgen receptor

The lead epitope was optimized by determining its interaction with androgen receptor using molecular docking. An androgen receptor is a specific receptor found on the cell surface of the host during infection of the MAGE-A11 antigen sequence. The ClusPro server was used to study the binding of the lead epitope with androgen protein receptor, using the same parameters given above.

### Peptide dynamics of lead epitope

CABS-dock coarse-grained molecular dynamics approach was employed for large-scale conformational epitope analysis. The structural changes in target protein are essential considerations for molecular dynamics analysis [47–49]. CABS molecular dynamics analyze the full conformation flexibility of epitope peptide and large-scale flexibility analysis of target protein. CABS method has been widely employed in different applications for molecular dynamics studies, structural conformational studies, and the assessment of peptide dynamics involving the mechanism of interaction [50,51]. The stability of interaction of lead epitope with androgen receptor was assessed by determining the RMSD of the complex, atomic fluctuations by element analysis of interaction and contact maps of epitope–protein.

### Immune response simulation analysis

The immune response elicitation capacity of the antigenic determinant was examined for important immune response regulators T-, B-cells, memory cells, and other immunogenic cells. The immune response analysis of the antigenic determinant was performed using the immune simulation method through C-ImmSim 10.1 server [52]. It is a potential algorithm to determine the immune response capability of the antigens by utilizing the machine learning-based mesoscopic scale simulator approach.

### Epitope stability optimization and population coverage analysis

The physicochemical properties of the lead epitope were analyzed to assess the conformational structural stability of epitope structure. The structural and conformational properties of the epitope were investigated by the ProtParam and preLOCFMD servers [53]. Stereochemical properties were assessed through the Ramachandran plot. Besides, the world population coverage of the lead epitope was also evaluated. This checks the population coverage and the authenticity of the predicted epitope to be used as a potent vaccine candidate. To identify the population coverage of the epitope, the antigenic sequences of epitope was submitted to the population coverage calculation tool of the IEDB [54].

### *In silico* cloning of lead epitope

*In silico* cloning was performed using the Codon usage wrangler (http://www.mrc-lmb.cam.ac.uk/ms/methods/codon.html). This server works by reverse translation and codon optimization of target epitopes for cloning and expression in a suitable vector. Codon usage wrangler server gives the cDNA sequence that is assessed for codon optimization and GC content by Genescript rare codon analysis server. GC content of a target sequence should come in a range of 30–70%. If the GC content value of the lead sequence does not lie in this range, it will result in undesired effects on transcriptional and translational regulation.

## Discussion

In the last few years, many clinical trials for cancer vaccine employing the antigenic peptides have been undertaken and shown positive impacts [55,56]. The approval of a therapeutic cancer vaccine has paved the way to design and develop the next generation highly specific vaccine with enhanced antitumor potency. A detailed understanding of the host target and tumor vaccine candidate interaction and the binding mechanism was required to design the cancer vaccine [57]. Identification of the short immune signature (epitopes) out of a sizeable carcinogenic protein has established an advanced approach to develop cancer vaccine therapeutics. The efforts have been made to identify the antigenic peptides for tumor-associated antigens and their interaction with the HLA molecules to derive substantial cancer vaccines [58]. The carcinogenic protein MAGE-A family is well known to play a critical role in regulations of cancer development and has been widely studied to design cancer immunotherapeutics. Among the various class of MAGE-A family, MAGE-A11 is a new class of tumor-associated antigen and reported to possess a direct role in immune modulation and consequent tumor development and progression. However, there are no reports for CTL-based epitope prediction for MAGE-A11 and leading that study to anticancer vaccine candidature to date as per the best of our knowledge.

Although, there are certain reports on MAGE-A family proteins (MAGE-A3 and MAGE-A24), other than MAGE-A11, but they were associated with a lack of potential immunological parameters and binding specificity to direct membrane receptors like with androgen receptor [59,60]. As it has been suggested in many studies, to incorporate the essential immunological parameters, including the antigenicity, immunogenicity, and TAP affinity along with HLA binding affinity to design the potential MAGE-CTL based vaccine [61,62]. Moreover, there are also no reports where large scale flexibility analysis was carried out, as it is well known that stable binding of CTL epitopes with membrane receptors can evoke a robust and prolonged immune response in melanoma patients [63].

Overcoming these deficiencies in the present study, the novel epitope for MAGE-A11 was designed by using the high throughput advanced immunoinformatics approaches. The study was highly focussed on the advanced strategy: first, to determine the top-scored CTL epitope from five different algorithms and common consensus epitopes out of them were identified. This way, we were able to identify the diverse, highly specific, and more authentic epitopes. Eight common consensus epitopes were identified. The second step was the assessment of the shortlisted epitopes through the potential immunological parameters (Antigenicity, C terminal proteasomal efficiency, TAP affinity, and high immunogenicity). The identified eight common consensus epitopes were further shortlisted using immunological properties, which showed the ‘KIIDLVHLL’ epitope was found to possess the high antigenic properties and potency with a high TAP affinity of 2039.65 nm (IC_50_ value).

The third vital step was to assess the binding affinity of designed vaccine candidate with their membrane specific androgen receptor along with the HLA receptor. The lead epitope (KIIDLVHLL) was evaluated for its binding with the HLA-A*0201protein and androgen receptor. HLA-A*0201 receptor binding epitopes are reported to trigger the high levels of functional cytotoxic T-cell response and, consequently, binding of MAGE-A11 antigens with androgen receptors to promote transcriptional activity and tumor progression in humans [64]. The results showed the strong binding of lead epitope with HLA-A*0201 (binding score −780.06 kcal/mol) and with androgen receptor with a binding score of −641.36 kcal/mol and involvement of five hydrogen bonds along with strong hydrophobic interactions. The fourth step of our study was highly significant, which employed CABS coarse-grained-based simulation approach. It is an implicit approach for large-scale protein rearrangements while explicating the peptide docking [65] since conventional molecular dynamic simulation analysis was restricted with rearrangement and flexible binding of antigenic peptide and ablation of flexibility to a target receptor. The conformational stability of the complex trajectory (lead epitope-receptor) was assessed and depicted a stable interaction with an average RMSD of 2.21 Å and the maximum RMSD value of 6.48 Å with a cluster density of 57.418. A large number of elements (127) were found to involve in the binding of cluster-1 (lead epitope) to the receptor through the energy minimized all-atom refinement analysis and contact map analysis. Prolonged stable binding of the lead vaccine candidate with the specific receptor by MD simulation run can be explained by the time required to evoke the effector immune response.

Moreover, the epitope’s world population coverage assessment showed to cover 39.08% of the total world population as per the IEDB database of the vaccine. With these affirmative outcomes, epitope sequence ‘KIIDLVHLL’ of the MAGE-A11 tumorogenic protein has been reported as a potential candidate for vaccine design with broad population coverage. With all these significant results through the advanced immunotherapeutic approach, we report the highly efficient cancer vaccine candidate. The designed CTL vaccine candidate for MAGE-A11 further needs to be studied through *in vitro* and *in vivo* assays. The reported CTL peptide can be used directly to elicit the CTL-specific melanoma responses and to stimulate the *in vitro* adoptive immunotherapy.

## Conclusion

MAGE proteins are reported for its restricted expression to reproductive tissues and express aberrantly in cancer states, hence antigenic determinant for MAGE-A11 carcinogenic protein has been determined by employing advanced immunological parameters, including the five different algorithms integrated with molecular dynamics studies. Resulting (9-mer: KIIDLVHLL) epitope can be used as a vaccine candidate and would be helpful to boost the immune system to fight against the growth and progression of tumor antigens. The antigenic CTL epitope showed the strong binding with HLA-A*0201 receptor protein and with androgen receptor (specific receptor for MAGE-A11 during infection) with high molecular docking scores. Also, the large-scale conformational rearrangement analysis of epitope and androgen receptor through molecular dynamics simulation studies showed the stable binding to the membrane receptor with minimum average RMSD of 2.21 Å and involvement of a large number of elements (127) by energy minimized all-atom refinement analysis and contact map analysis. The lead epitope has high antigenicity and immunogenicity characteristics along with high population coverage, which ensured the induction of high immune response in the host. In conclusion, the present study reports the CTL-based as a highly potential cancer vaccine candidate.

## Abbreviations

CTL: cytotoxic T lymphocyte
IEDB: Immune Epitope Database
MAGE: melanoma antigen
MHC: major histocompatibility complex
PDB: Protein Data Bank
Rg: radius of gyration
RMSD: root mean square deviation
TAP: transporter associated with antigen processing
